# A Novel Fixation System for Acetabular Quadrilateral Plate Fracture: A Comparative Biomechanical Study

**DOI:** 10.1155/2015/391032

**Published:** 2015-01-31

**Authors:** Guo-Chun Zha, Jun-Ying Sun, Sheng-Jie Dong, Wen Zhang, Zong-Ping Luo

**Affiliations:** ^1^Orthopaedic Department, The First Affiliated Hospital of Soochow University, 188 Shizi Street, Suzhou, Jiangsu 215006, China; ^2^Department of Orthopedic Surgery, Affiliated Hospital of Xuzhou Medical College, Xuzhou, Jiangsu 221002, China; ^3^Orthopedic Institute, Soochow University, 708 Renmin Road, Suzhou, Jiangsu 215006, China

## Abstract

This study aims to assess the biomechanical properties of a novel fixation system (named AFRIF) and to compare it with other five different fixation techniques for quadrilateral plate fractures. This in vitro biomechanical experiment has shown that the multidirectional titanium fixation (MTF) and pelvic brim long screws fixation (PBSF) provided the strongest fixation for quadrilateral plate fracture; the better biomechanical performance of the AFRIF compared with the T-shaped plate fixation (TPF), L-shaped plate fixation (LPF), and H-shaped plate fixation (HPF); AFRIF gives reasonable stability of treatment for quadrilateral plate fracture and may offer a better solution for comminuted quadrilateral plate fractures or free floating medial wall fracture and be reliable in preventing protrusion of femoral head.

## 1. Introduction

Despite improvement in surgical technique, acetabular fracture involving quadrilateral plate represents a challenge to the surgeon because of its deep location and a very thin bone [1–4], especially in elderly individuals which are often combined with comminution and osteoporosis [4, 5]. To achieve anatomic reduction of the fracture, a stable fixation, and preventing protrusion of the femoral head is imperative for optimal outcome [5–8].

Currently, a variety of implants are available for displaced quadrilateral plate fractures, including the cerclage wire-plate composite [1], pelvic brim long screws [6], various combinations of spring plates (T-shaped plate [9], L-shaped plate [4], infrapectineal plate [10], H-plate [6], and multidirectional titanium fixator [6]). These devices are not entirely without any limitation [4], such as requiring great surgical skills and experience, a high risk of joint penetration, or inadequate fixation of the comminuted segments, particularly in fractures with osteoporotic bones, which may be associated with inadequate reduction and instability of fixation leading to joint incongruity and early arthritis.

In view of these concerns, we designed a novel fixation system (named acetabular fracture reduction internal fixator, AFRIF (number ZL200810024632.6, CN PAT)) for acetabular fractures involving the quadrilateral plate. Clinically, we have successfully used the AFRIF for comminuted quadrilateral plate fractures in some patients, but it has never been tested previously for the biomechanical features. Therefore, the purpose of this study was to discuss the AFRIF's design principle and evaluate the biomechanical properties of the AFRIF and to compare it with other five different fixation techniques for quadrilateral plate fractures.

## 2. Materials and Methods

### 2.1. Design of the AFRIF

**Figure 1:**
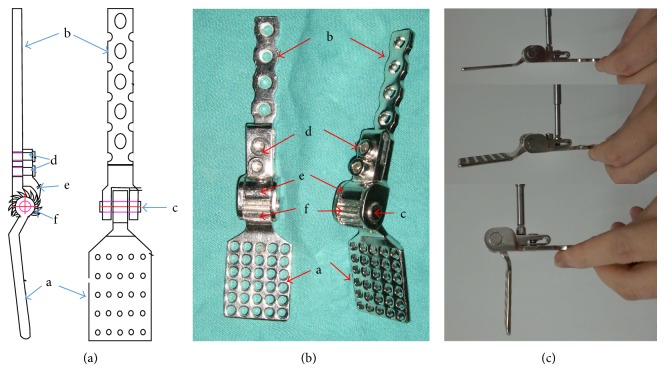
(a)–(c) Illustration and photograph demonstrating all components of the AFRIF ((a) and (b)): a: a segmental plate of inner wall; b: a segmental plate of ilium; c: a connecting arm; d: a set bolt; e: a pawl; f: a ratchet. (c) The photograph demonstrating that a pawl can cooperate with the ratchet and the set bolt, which formed unidirectional articulated joint from medial to lateral.

The novel fixation system consists of five parts (Figures 1(a) and 1(b)): (1) a segmental plate of inner wall. The plate is designed in terms of the shape and dimensions of quadrilateral plate, which is expanded to hold the quadrilateral plate. The proximal portion of the plate has an extended arm with a ratchet; (2) a segmental plate of ilium. The distal portion of the plate has a space which connects the ratchet; (3) a connecting arm that links the ratchet to the segmental plate of ilium, which formed an articulated joint; (4) a set bolt; (5) a pawl that can cooperate with the ratchet and the set bolt, forming unidirectional articulated joint from medial to lateral. When the bolt is tightened, the pawl engages in the ratchet, which can control the angle between the segmental plate of the inner wall and segmental plate of ilium and keep reduction and immobilization for quadrilateral plate fractures (Figure 1(c)).

### 2.2. Operative Technique

**Figure 2:**
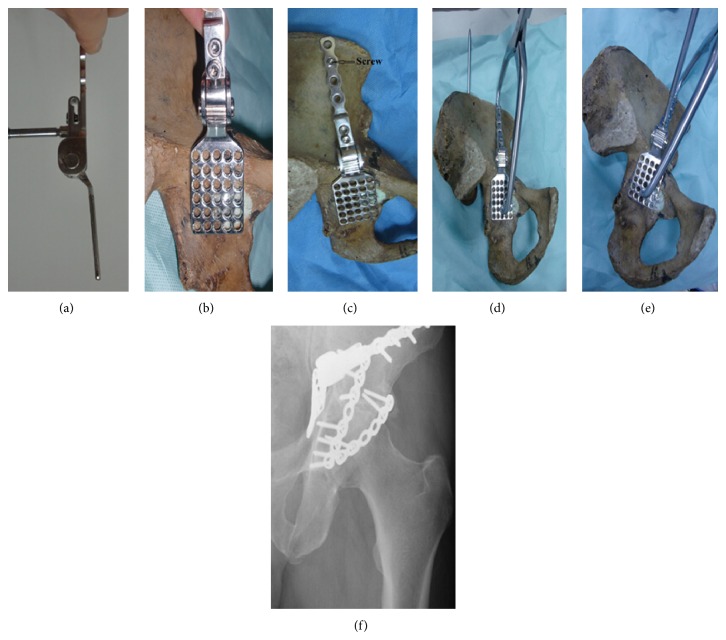
(a)–(f) The operative procedure of the AFRIF technique for quadrilateral plate fracture. First the set bolt is loosened (a); then the segmental plates of inner wall (b) and ilium (c) were placed in the appropriate location of quadrilateral plate and the ilium, respectively. The segmental plate of ilium was fixed first (c), and then a Mayo clamp is placed from the anterior inferior iliac spine to an appropriate hole in the segmental plate of inner wall which acts as a direct reduction force on the quadrilateral plate (d). After obtaining anatomical reduction of the quadrilateral plate fracture, the set bolt of the AFRIF was tightened (e) to gain a fixed angle between the segmental plate of inner wall and ilium in which the reduction can be maintained. (f) Postoperative X-ray shows that the quadrilateral plate fracture was fixed by the AFRIF.

First the set bolt is loosened; then the segmental plates of the inner wall and ilium were placed at the appropriate location of quadrilateral plate and ilium, respectively. The segmental plate of the ilium was fixed first; then a Mayo clamp is placed from the anterior inferior iliac spine to an appropriate hole on the segmental plate of inner wall which acts as a direct reduction force on the quadrilateral plate. After obtaining anatomical reduction of the quadrilateral plate fracture, the set bolt of the AFRIF was tightened to gain a fixed angle between the segmental plate of inner wall and ilium in which the reduction can be maintained (Figure 2). The AFRIF, unlike the traditional fixation techniques, can assist in maintaining the reduction and therefore may prevent against secondary loss of reduction during the subsequent fixation.

### 2.3. Specimens

Ten fourth-generation synthetic hemi-pelvises (Hemi-pelvis 3405, Sawbones, Pacific Research Laboratories, Vashon, WA, USA) were used.

### 2.4. Fracture Model

Isolated quadrilateral plate fracture was surgically created by using a band saw with a 0.5 mm blade, according to Culemann et al. [6]. After fracture creation, fine-grained sandpaper was used to smooth the fracture surfaces before hardware application.

### 2.5. Testing Protocol

**Figure 3:**
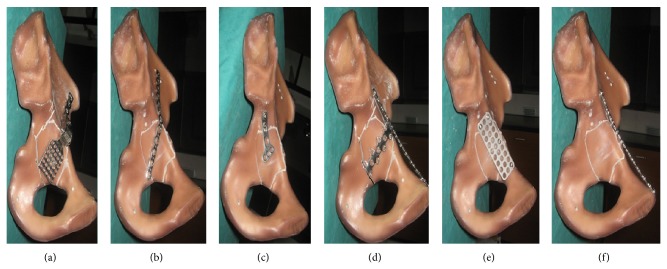
(a)–(f) Representative photograph of the different suture techniques: (a) AFRIF (AFRIF), (b) L-shaped plate fixation (LPF), (c) T-shaped plate fixation (TPF), (d) H-shaped plate fixation (HPF group), (e) multidirectional titanium fixation (MTF), and (f) pelvic brim long screws fixation (PBSF).

Initially, thirty synthetic hemi-pelvises have been purchased for the experiment and planned to use each fixation technique on five specimens. However, the preexperimental result showed that it was very difficult to prepare all fracture models in an identical fashion because of the complex acetabular morphology. In such situations, we found significant difference in the biomechanical properties, even if the same fixation technique was used for the fracture model. Therefore, the plan to test the biomechanics was changed so that each specimen was tested in six conditions (Figure 3): (1) AFRIF (AFRIF group), (2) L-shaped plate fixation (LPF group), (3) T-shaped plate fixation (TPF group), (4) H-shaped plate fixation (HPF group), (5) multidirectional titanium fixation (MTF group), and (6) pelvic brim long screws fixation (PBSF group). Originally, we intended to measure the biomechanical strength of infrapectineal plate, but the preexperimental result showed that it could not stabilize the quadrilateral plate which was separated from the anterior and posterior column which was the condition in our fracture model in this study.

**Figure 4:**
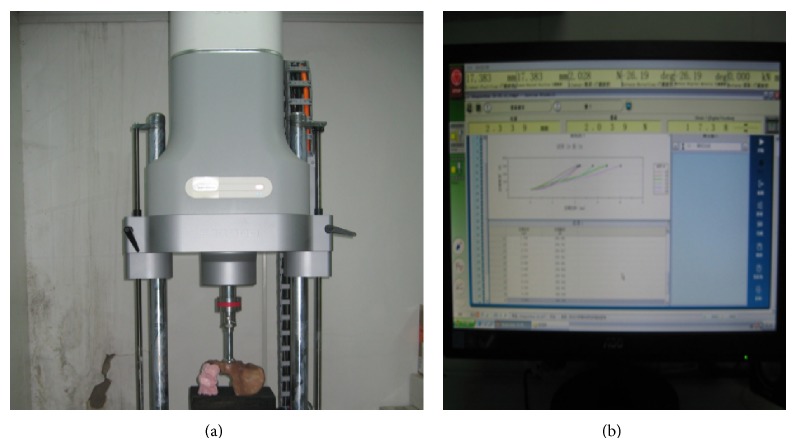
(a) and (b) Mechanical testing setup. (a) The universal material testing machine (Instron ElectroPuls E10000, Instron Corporation Norwood, MA, USA); (b) the microprocessor.

The synthetic hemi-pelvis was fixed rigidly to a customized fixture which was placed on the testing table of the universal material testing machine (Instron ElectroPuls E10000, Instron Corporation Norwood, MA, USA, Figure 4(a)), and we used a customized metal femoral head connected to the load cells of the universal material testing machine. The position of synthetic hemipelvis is the metal femoral head perpendicular to the acetabulum, by previously described protocol [11]. We chose the position to test the hemipelvis in such a way that the direction of the force facilitates adequate motion at the fracture site so that it could better reflect the strength of these implants.

The biomechanical properties were assessed using four factors: the load to create 2 mm displacement; the load to create 3 mm displacement, displacement under the maximum load level of 300 N, and stiffness. We chose the 2 mm and 3 mm as an evaluation factor because current literature suggests that fracture with >3 mm of residual displacement (poor reduction) in the general population or 2 to 3 mm of residual displacement (imperfect reduction) in younger patients can result in poor clinical outcomes [12–14]. A maximum load level of 300 N was chosen in consideration of the fact that the maximum load withstood is only 20 kg for those patients within 3 months of the open reduction and internal fixation [15].

First, we compared the difference of load to create 2 mm and 3 mm displacement with six different fixation techniques. Five specimens and six implants were used at this stage. Each specimen was subsequently fixed using six implants and was successively measured for the load. Later this procedure is carried out on the remaining specimens. The acetabulum was continuously loaded to create 4 mm displacement or until failure of the fixation construct (5 N/s) that was defined as a change in the load-displacement curve. The course of the loading during displacement was autorecorded via the microprocessor (Figure 4(b)) output of the universal material testing machine.

Second, other five specimens were tested to compare the difference of displacement under the maximum load level of 300 N in the six different fixation techniques by the above mentioned procedure. The acetabulum was continuously loaded to 300 N or until failure of the fixation construct (5 N/s). The course of the displacement under the loading was autorecorded via the microprocessor output of the universal material testing machine.

### 2.6. Statistical Analysis

All analyses were performed using Stata version 12.0 for Windows (StataCorp LP, College Station, TX). The mechanical parameters were presented as mean ± standard deviation. A Kolmogorov-Smirnov test for normal distribution and Bartlett test for homogeneity of variances were used before further statistical analysis for quantitative data was conducted. The analysis of variance (ANOVA) was performed when the measurement data were able to meet the parametric assumptions. The level of significance was set at *α* = 0.05. A value of *P* < 0.05 was considered statistically significant.

## 3. Results

### 3.1. The Load Applied to Create 2 mm and 3 mm Displacement

**Figure 5:**
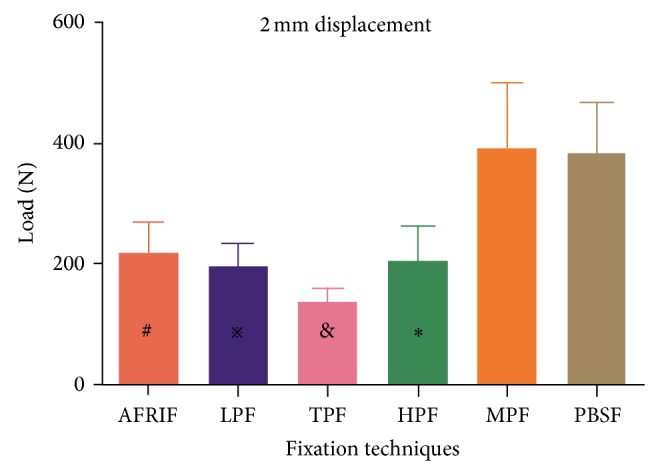
Comparison of mean load applied to create 2 mm of displacement. ^#^Significant difference compared with TPF, MPF, and PBSF (*P* = 0.001, *P* = 0.001, and *P* = 0.001, resp.). ^*※*^Significant difference compared with TPF, MPF, and PBSF (*P* = 0.005, *P* = 0.000, and *P* = 0.000, resp.). ^&^Significant difference compared with HPF, MPF, and PBSF (*P* = 0.005, *P* = 0.001, and *P* = 0.001, resp.).  ^*^Significant difference compared with MPF and PBSF (*P* = 0.000 and *P* = 0.000, resp.).

**Figure 6:**
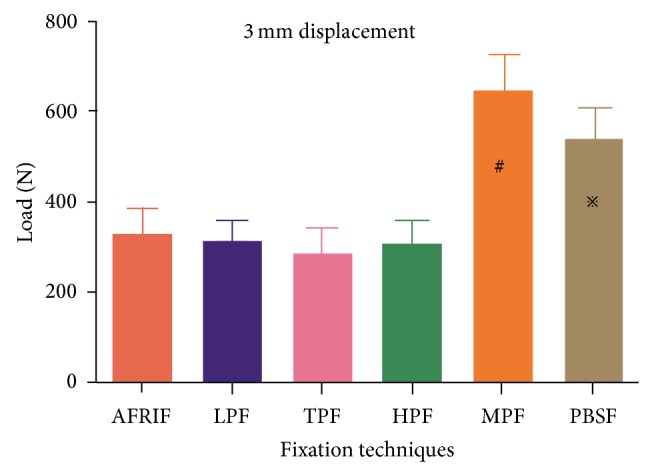
Comparison of mean load applied to create 3 mm of displacement. ^#^Significant difference compared with AFRIF, LPF, TPF, and HPF (*P* = 0.000, *P* = 0.000, and *P* = 0.000 and *P* = 0.000, resp.). ^*※*^Significant difference compared with AFRIF, LPF, TPF, and HPF (*P* = 0.001, *P* = 0.000, *P* = 0.000, and *P* = 0.000, resp.).

The group data for mean load to create 2 mm or 3 mm displacement are shown in Table 1. The PBSF and MTF group showed significantly higher mean load at 2 mm and 3 mm displacement compared with the AFRIF, LPF, TPF, and HPF group (*P* < 0.05) (Figures 5 and 6). No significant differences were found between the PBSF and MTF group, as well as between the AFRIF, LPF, and HPF group (*P* > 0.05). At 2 mm displacement, the lowest load was found for the TPF group, with significant differences compared with all other groups (*P* < 0.05) (Figure 5).

### 3.2. Displacement under the Maximum Load Level of 300 N

**Figure 7:**
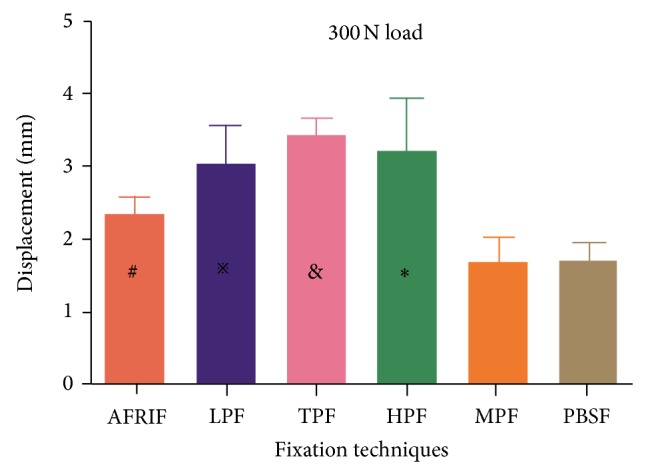
Comparison of mean displacement under the maximum load level of 300 N. ^#^Significant difference compared with LPF, TPF, HPF, MPF, and PBSF (*P* = 0.009, *P* = 0.000, *P* = 0.003, *P* = 0.011, and *P* = 0.009, resp.). ^*※*^Significant difference compared with MPF and PBSF (*P* = 0.000 and *P* = 0.000, resp.). ^&^Significant difference compared with MPF and PBSF (*P* = 0.000 and *P* = 0.000, resp.).  ^*^Significant difference compared with MPF and PBSF (*P* = 0.000 and *P* = 0.000, resp.).

Table 2 shows the group results for displacement when the loading achieved intensity of 300 N. When specimens were loaded to 300 N, compared with all the other groups, the PBSF and MTF group showed the minimum displacement (*P* < 0.05) and the AFRIF group showed the second minimum one (*P* < 0.05) (Figure 7). No significant differences were found between the PBSF and MTF group (*P* > 0.05), as well as between the LPF, TPF, and HPF group (*P* > 0.05). No failure of the fixation construct was recorded during loading of the specimens.

### 3.3. Stiffness

**Figure 8:**
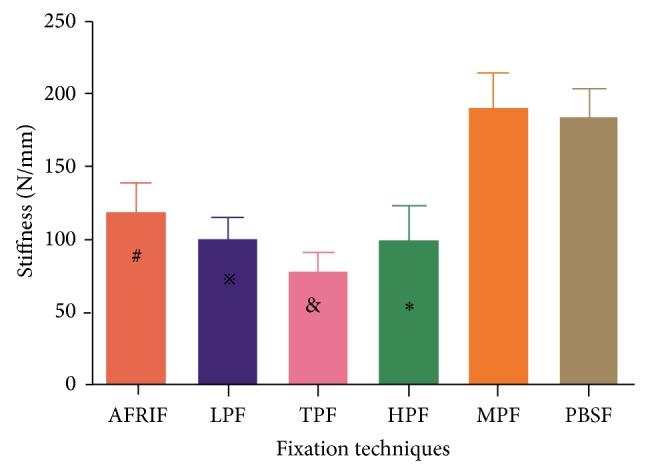
Comparison of mean stiffness. ^#^Significant difference compared with LPF, TPF, HPF, MPF, and PBSF (*P* = 0.009, *P* = 0.000, *P* = 0.009, *P* = 0.000, and *P* = 0.000, resp.). ^*※*^Significant difference compared with TPF, MPF, and PBSF (*P* = 0.005, *P* = 0.000, and *P* = 0.000, resp.). ^&^Significant difference compared with HPF, MPF, and PBSF (*P* = 0.005 and *P* = 0.000, resp.).  ^*^Significant difference compared with MPF and PBSF (*P* = 0.000 and *P* = 0.000, resp.).

Table 2 shows the group results for stiffness. The MTF and PBSF group showed the highest values among all other groups (*P* < 0.05), the AFRIF group showed the second highest values among the remaining groups (*P* < 0.05), and the TPF group has the minimum values (*P* < 0.05) (Figure 8). No significant differences were found between the MTF and PBSF group (*P* > 0.05), as well as between the LPF and HPF group (*P* > 0.05) (Figure 8).

## 4. Discussion

The incidence of acetabular fractures in the elderly is on the rise with an ageing population typically in modern society [5]. Among the elderly, acetabular fractures are most commonly combined with quadrilateral plate fractures [5]. Some quadrilateral plate fractures can be reduced indirectly along with the reduction and stabilization of column fractures [4]; while this methods of reduction is unavailable to the comminuted quadrilateral plate fracture and the free floating medial wall fracture. Concerning the limitations to achieve a stable fixation in that condition, we designed the AFRIF for the quadrilateral plate fractures with comminuted or free floating medial wall fracture. Though this fixation technique had been successfully used to treat some patients, we were interested in comparing it with other five different fixation techniques for quadrilateral plate fractures.

This biomechanical test shows that the mean load to create fracture displacement of 2 mm and 3 mm in AFRIF group was 220.2 ± 49.0 N and 327.9 ± 52.3 N, respectively. The mean stiffness was 119.9 ± 21.1 N/mm. Considering the load of acetabulum in patient which was around 20 kg after the surgery within 3 months, the strength of AFRIF may be lower. However, we believe that AFRIF can provide a reasonable stability of treatment for quadrilateral plate fracture because of the method to measure the strength of AFRIF in this study which was measured under loading of the direct force in the perpendicular direction. Nevertheless, actually the force exerting the acetabulum falls into two categories: the horizontal force and the vertical one [16].

The MTF and PBSF fixation techniques for quadrilateral plate fracture have been demonstrated to have more biomechanical stability by this and previous biomechanical study [6]. However, the MTF for quadrilateral plate fracture may use a Stoppa approach, precaution is to be taken as critical anatomic structures such as corona mortis pass through the surgical field [2]. Therefore, choosing this surgical approach, the learning curve is quite sharp and long and has a high risk of bleeding and morbidity if the corona mortis is injured [2]. In addition, the biomechanical strength of the MIF may decrease in the presence of an osteoporotic bone, because that is dependent on the holding power of a screw which is associated with the quality of bone. It is previously reported that the PBSF was gold standard to stabilize quadrilateral plate fracture and has biomechanical advantages [6, 17]; however, the placement of these screws has been associated with high risk of joint penetration and failed to fix and maintain reduction of the fracture due to its comminution [4, 16–19]. This problem also was observed in our preliminary experiment which showed 2 of 6 screws penetrate the joint surface.

The AFRIF fixation technique provided a stronger fixation than TPF, LPF, and HPF. In order to avoid the risk of intra-articular penetration of screw, the LPF, HPF, and TPF fixation techniques have been used to stabilize the quadrilateral plate fracture [4, 6, 9]. However, prebending of LPF and HPF may be difficult to obtain an accurate contour to buttress the fragments because of the irregular profile of the outer surface of the acetabulum, which may influence the stability of the fracture, leading to nonunion and the need for secondary arthroplasty [4, 6]. In addition, all of the LPF, HPF, and TPF fixation techniques may be limited to simple large medial wall fracture fragment [4, 9], which for comminuted quadrilateral plate fractures may result in instable fixation and inadequate reduction that leave residual protrusion of the femoral head and hip incongruence which leads to early arthritis. Clinically, we found that the AFRIF has the following advantages:.(1) There is no need for an additional procedure for the prebending of the implant during the operation, which greatly simplified the operation. (2) It has the ability to assist in maintaining the reduction at the time of fixation which can be protective against secondary loss of reduction during the subsequent fixation. (3) The segmental plate of inner wall can hold the comminuted fracture segments and maintain stable fixation, which can effectively prevent displacement of the medial wall and protrusion of the femoral head. Hence the AFRIF may be particularly suitable for severely comminuted fractures. (4) The screw is not employed to fix the quadrilateral plate, therefore posing no risk of articular penetration. (5) The AFRIF treatment for quadrilateral plate fracture can be accomplished through the lateral two of the three windows of ilioinguinal approach [20]. This more limited exposure may save operative time and reduce blood loss.

Several limitations to this study are noted. First, we only used synthetic hemi-pelvises because many authors think it can minimize the effect of confounding variables and guarantees a better reproducibility [6, 10, 21–23]. Second, fracture model was using quadrilateral plate fractures alone, which may not be able to demonstrate the mode of fracture seen in clinical practice. The results may be different if the fixation techniques could be loaded in a more clinical condition. However, many acetabular fracture types combined with quadrilateral plate injury, such as anterior column, anterior with posterior hemitransverse, associated both columns, and anterior wall [5]; it may be difficult to simulate all types of fractures combined with quadrilateral plate injury and use these models to test the biomechanical properties of the six fixation techniques. In addition, the six fixation techniques are mainly used to hold quadrilateral plate fractures instead of the other part of acetabular fracture. Therefore, we have evaluated the stability only in quadrilateral plate fractures alone. Third, we did not simulate a physiologic loading condition which is cyclic in nature; therefore we were unable to evaluate fatigue failure of these six fixation techniques under the cyclic loading. But the authors feel that static loading allows for the comparison of biomechanical properties of implants during the early stages of healing, and biomechanical stabilities have an important influence on fracture healing during this period. Therefore, this study can provide biomechanical guidelines for selecting implants for quadrilateral plate fracture. Fourth, we did not simulate an osteoporotic fracture model. Acetabular fracture involving quadrilateral plate occurs more frequently in the elderly patients with osteoporosis. Therefore, the results of this study may not reflect fixation strength of these implants for quadrilateral plate fracture in elderly patients, especially in the TPF, MTF, and PBSF, in which stability of the fixation depends on quality of bone. Fifth, one model was reused for six fixation techniques, which may affect the assessment of biomechanical strength for these implants. But they are not fixed at the same focal point, so in theory this does not affect the biomechanical characteristics of the implant.

## 5. Conclusions

In summary, this in vitro biomechanical experiment has shown that the MTF and PBSF provided the strongest fixation for quadrilateral plate fracture. The better biomechanical performance of the AFRIF compared with the TPF, LPF, and HPF; AFRIF gives reasonable stability of treatment for quadrilateral plate fracture and may offer a better solution for comminuted quadrilateral plate fractures or free floating medial wall fracture and be reliable in preventing protrusion of femoral head.

## Figures and Tables

**Table 1:** The load applied to create 2 mm and 3 mm of displacement (mean ± SD).

Fixation technique	Load (N) to create	
	2 mm displacement	3 mm displacement
AFRIF	220.2 ± 49.0	327.9 ± 52.3
LPF	199.8 ± 34.9	310.0 ± 46.0
TPF	138.4 ± 20.7	284.4 ± 61.0
HPF	206.6 ± 56.4	306.3 ± 48.1
MTF	395.2 ± 107.7	645.7 ± 77.8
PBSF	386.0 ± 83.0	539.2 ± 68.6

**Table 2:** Displacement under the maximum load level of 300 N and stiffness (mean ± SD).

Fixation technique	Displacement (mm)	Stiffness (N/mm)
AFRIF	2.3 ± 0.2	119.9 ± 21.1
LPF	3.0 ± 0.5	100.8 ± 15.5
TPF	3.4 ± 0.2	78.5 ± 12.6
HPF	3.2 ± 0.7	100.4 ± 24.3
MTF	1.7 ± 0.3	192.1 ± 43.1
PBSF	1.7 ± 0.3	187.0 ± 33.8
